# Interleukine-6 and TNFα blockers provide only partial and short-term temporal improvement in cryopyrin-associated periodic syndrome

**DOI:** 10.1186/1546-0096-12-S1-P271

**Published:** 2014-09-17

**Authors:** Vladimir Dolgikh, Elena Kalashnikova, Ludmila Snegireva, Lubov Rychkova, Anna Pogodina, Tatyana Knyazeva, Mikhail M Kostik

**Affiliations:** 1 Scientific Center of Family Health and Human Reproduction problems. Siberian Branch of the Russian Academy of Medical Sciences, Irkutsk, Russian Federation; 2 Perm Regional Clinical Hospital, Perm, Russian Federation; 3 Saint-Petersburg State Pediatric Medical University, Saint-Petersburg, Russian Federation

## Introduction

Cryopyrin-associated periodic syndrome (CAPS) is an inherited disease which is caused by gain-of function mutations in CIAS1 gene function resulting in increased secretion of active IL-1β. IL-1β also can stimulate the production of other proinflammatory cytokines, such as IL-6, TNFα and so on (downstream cascade). The use of IL-1 blockers dramatically controls inflammation in CAPS patients and provide a persistent amelioration of the inflammatory manifestations. The data about using of IL-6 or TNFα blockers in CAPS are very limited.

## Objectives

The aim of our retrospective study was to describe the effects of IL-6 and TNFα blockers in CINCA/NOMID syndrome.

## Methods

We describe the effects of IL-6 and TNFα blockers in 4 patients with CINCA/NOMID syndrome who were treated in their local hospitals by tocilizumab (n=2), infliximab (n=1) and adalimumab (n=1). Patient#1 (Pt1) with CINCA/NOMID was treated by TCZ due to lack of access to IL-1 blockers, Pt2 was misdiagnosed as systemic JIA and received TCZ, Pt3 was misdiagnosed as Crohn’s disease and was treated by the infliximab and Pt4 was misdiagnosed as a TRAPS and was treated by adalimumab. All patients had concomitant corticosteroids (CS).

## Results

In Pt1 an initial clinical and laboratory response was observed after the first infusion (no fever, no rash). However 2 week after the first infusion the patients had a relapse of the disease. . The second infusion was less effective, leading to a partial clinical response with persistent elevation of acute phase reactants (CRP, ESR) and WBC. The third infusion was ineffective and associated with infusion reaction and TCZ was stopped.

In Pt2 TCZ was initiated after CS and methotrexate fall. During TCZ course fever and rash decreased but not disappeared, CRP, SAA and ESR were normalized but no changes in ear (senso-neural deafness), eye (conjunctivitis and uveitis) and brain damage (morning headaches with vomiting). Steroids were tapered but not discontinued due to flares of the disease.

In Pt3 two initial Infliximab infusions (every 2 weeks) were effective: no fever, no rash and normalized CRP and ESR. It was difficult to evaluate any changes in the organs damage due to small age of child. After 2^nd^ infliximab infusion Pt3 experienced the flare and 3^rd^ infliximab infusion was ineffective and discontinued. Steroids were tapered but not discontinued due to flare of the disease.

In Pt4 adalimumab (40 mg sc every 2 weeks) was ineffective: only short-term decreasing of CRP and ESR. Girl still had all features of the disease and really controlled by only CS. She had impressive linear growth delay, open fontanels, hydrocephaly, chronic meningitis and acute phase reactants.

Later Pt2, 3, 4 were assessed by authors of this abstract and the CINCA/NOMID syndrome was diagnosed by clinically (n=3) and genetically (n=2, 1-negative mutations). Canakinumab was initiated in all children and provided prompt anti-inflammatory effect.

## Conclusion

The failure of anti-IL-6 and anti-TNFα treatment in CAPS patients confirms the dominant role of IL-1 in the pathogenesis of the disease and only IL-1 blockers strongly needed for these patients.

## Disclosure of interest

None declared.

